# Supplementary material for: Characterization of Antibiotic-Resistance Antarctic Pseudomonas That Produce Bacteriocin-like Compounds
Source: Microorganisms. 2024 Mar 6;12(3):530. doi: 10.3390/microorganisms12030530 (PMC10974256; doi:10.3390/microorganisms12030530)
Supplement: Supplementary file 1 [file microorganisms-12-00530-s001.zip › microorganisms-2635345-supplementary.pdf]

C1-4-7 **D2-4-6** **M1-4-11** ***S. aureus*** ***P. aeruginosa***

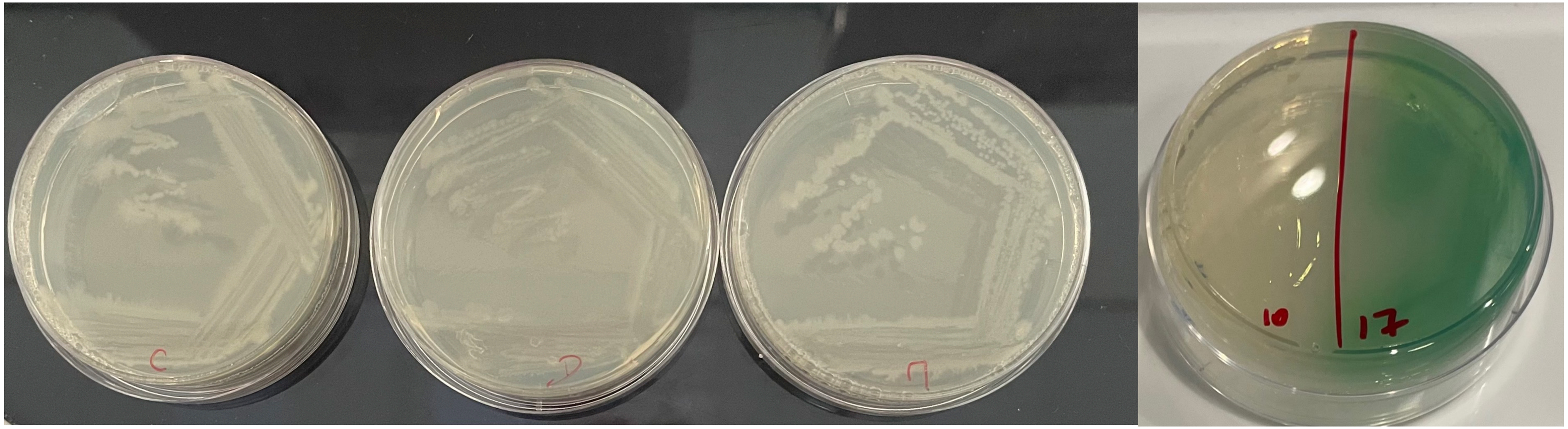

Figure S1. Growth of Antarctic isolates C1-4-7, -D2-4-6 and M1-4-11 on selective cetrimide agar medium for *Pseudomonas*. Positive control: *P. aeruginosa*. Negative control: *S. aureus*

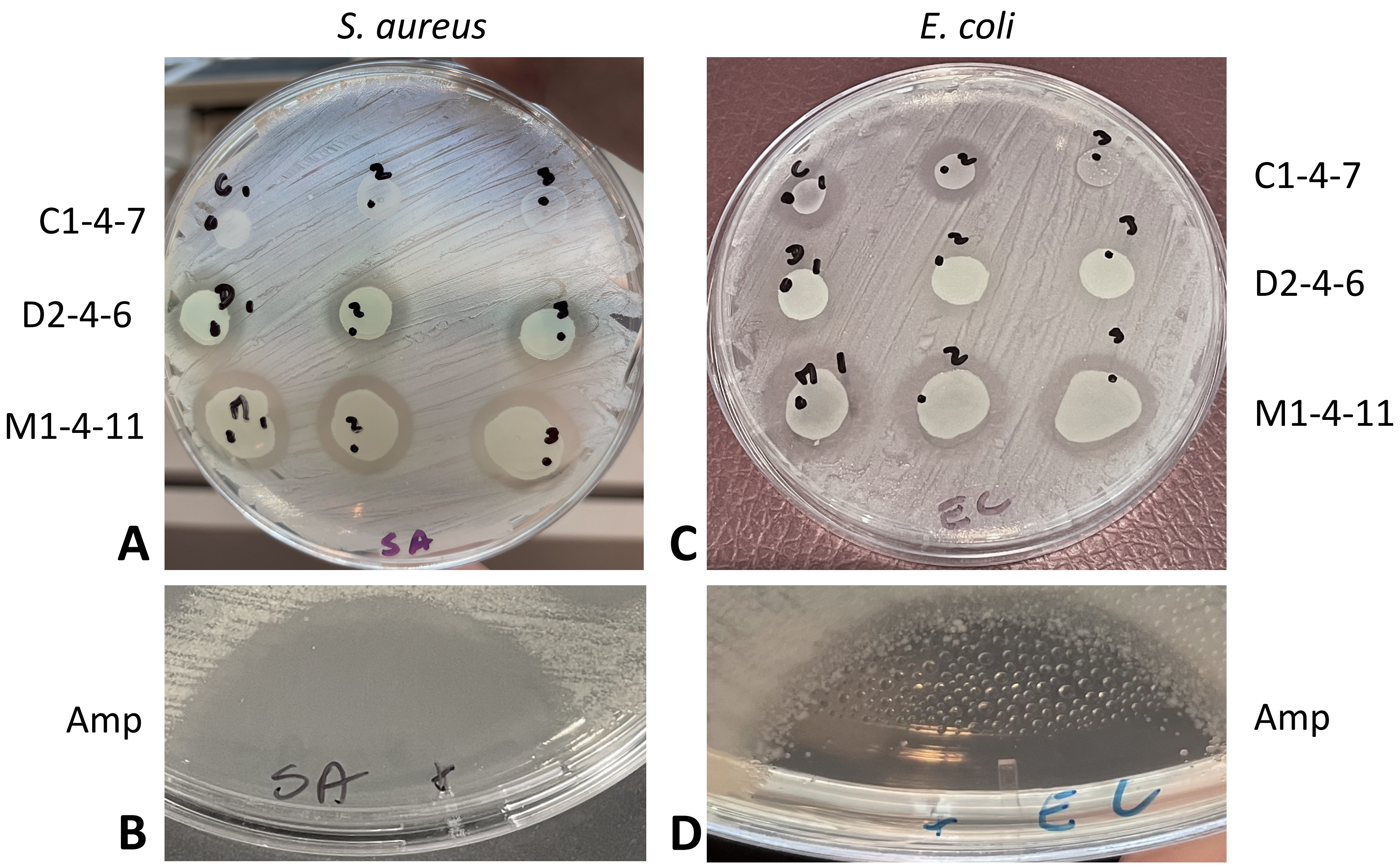

Figure S2. Example of antagonistic effect of Antarctic bacteria C1-4-7, D2-4-6 and M1-4-11 against reference bacterial pathogens **(A and B)** *S. aureus*, **(C and D)** *E. coli*. **(B and D)** Positive control: Ampicillin

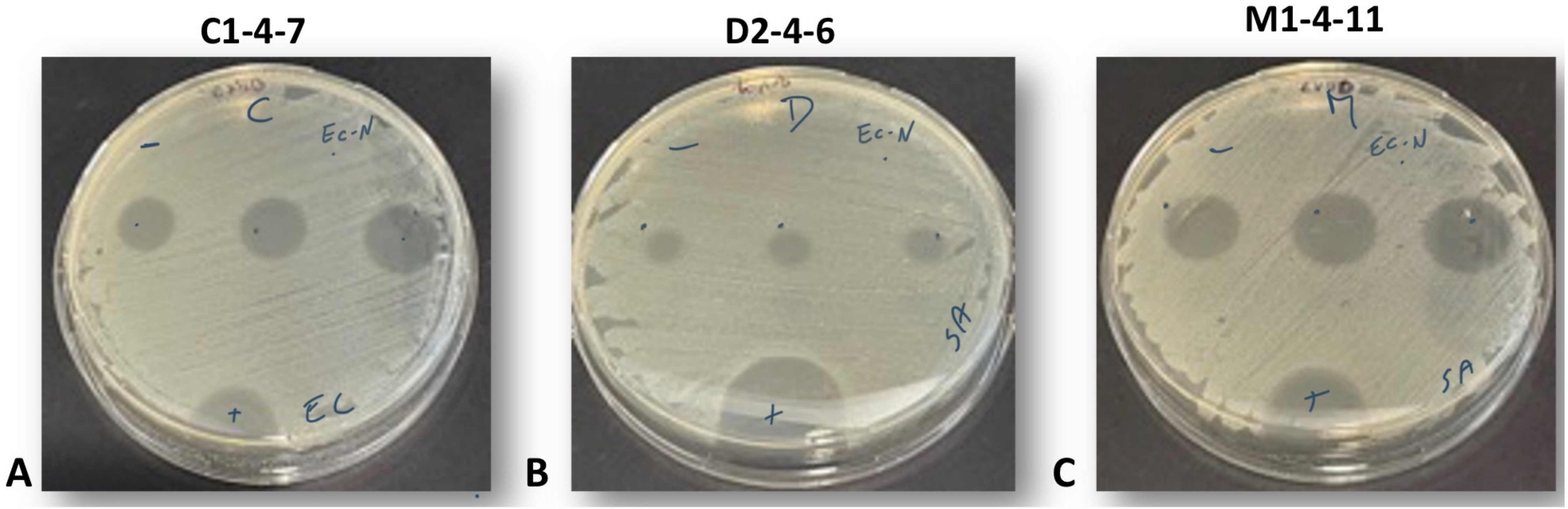

Figure S3. Example of antimicrobial activity of 20-fold concentrated supernatant of isolates **(A)** C1-4-7, **(B)** D2-4-6 and **(C)** M1-4-11 against reference bacterial pathogens (*S. aureus, E. coli*). Positive control: **(+)** Ampicillin. Negative control: **(-)** corresponding to 20-folds concentrated Nut1/3 and **EC-N** corresponding to 20-fold concentrated supernatant of *E. coli*. Indicator pathogen: **A**: *E. coli* (EC); **B** y **C**: *S. aureus* (SA).

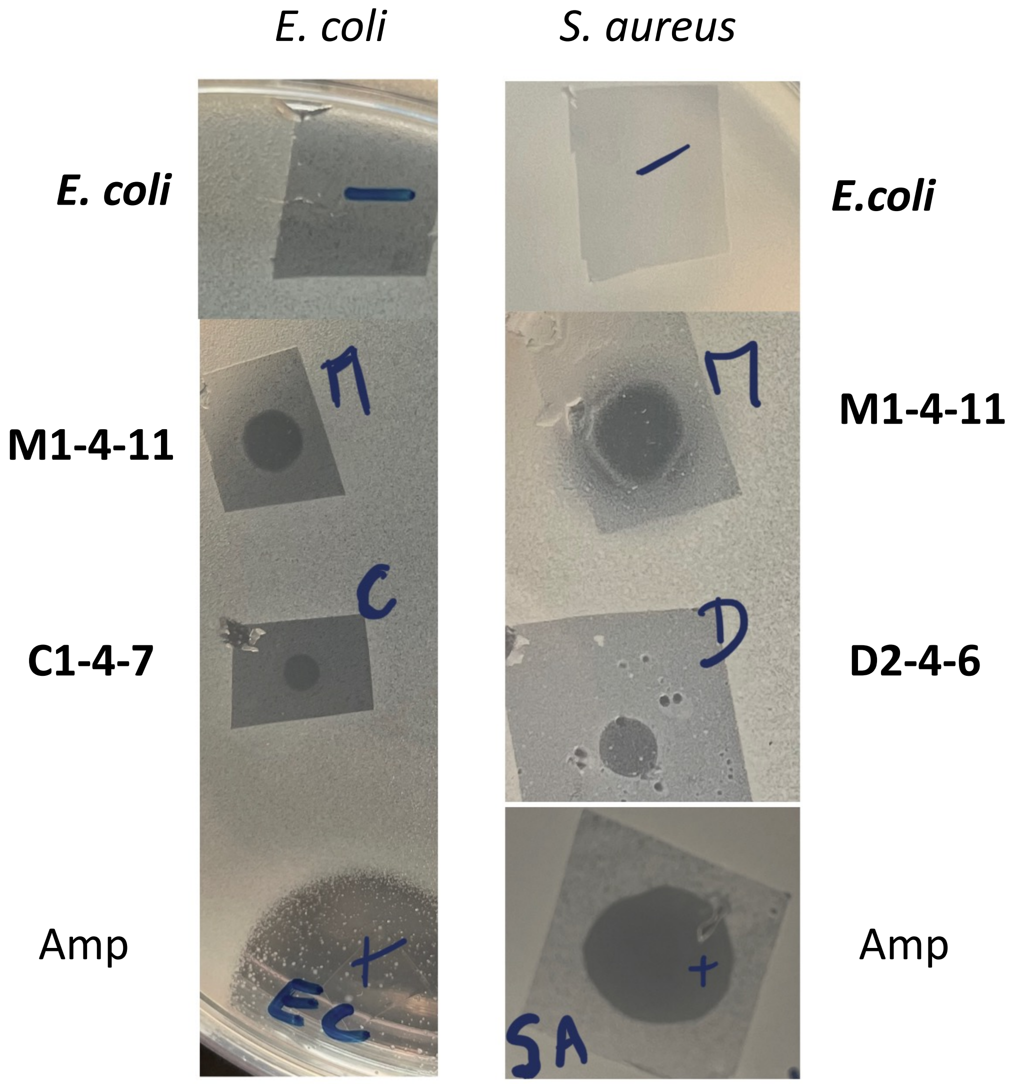

Figure S4. Antimicrobial activity of 20-fold concentrated supernatant of isolates C1-4-7, D2-4-6 and M1-4-11 via a 10kDa MWCO membrane. Reference bacterial pathogens: *S. aureus*, *E. coli*. Positive control: **(+)** Ampicillin. Negative controls: **(-)** 20-fold concentrated supernatant of *E. coli*.

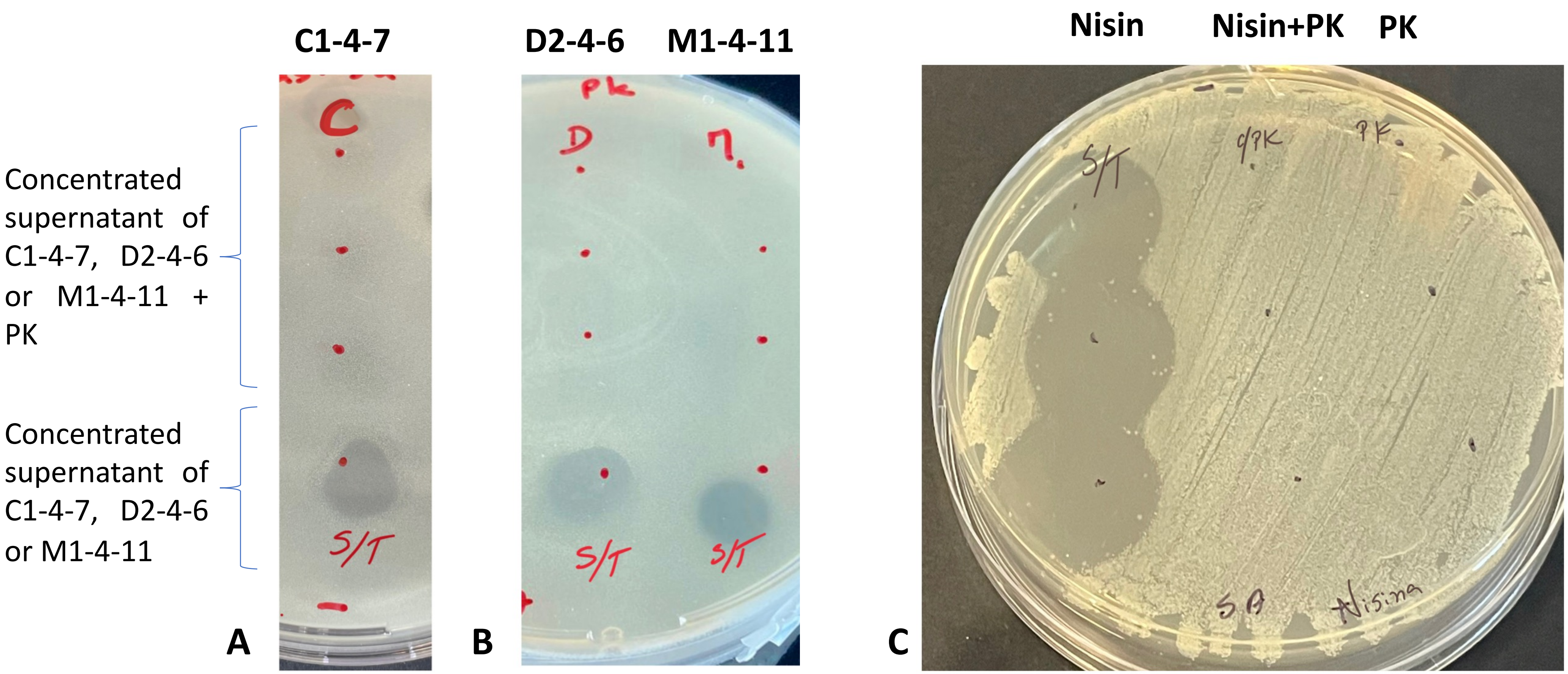

Figure S5. Effect of proteinase K (PK) on the antimicrobial activity of 20-fold concentrated supernatants of bacterial isolates C1-4-7, D2-4-6 and M1-4-11. Reference pathogenic bacteria **(A)** *E. coli*, **(B)** *S. aureus*. **(C)** Controls: Nisin + 20-fold Nut 1/3; Nisin + 20-fold Nut 1/3+PK; PK.
